# *Gluconobacter oxydans* DSM 50049 *–* an efficient biocatalyst for oxidation of 5-formyl-2-furancarboxylic acid (FFCA) to 2,5-furandicarboxylic acid (FDCA)

**DOI:** 10.1186/s12934-025-02689-x

**Published:** 2025-03-19

**Authors:** Mahmoud Sayed, Mohamed Ismail, Anirudh Sivasubramanian, Riko Kawano, Chengsi Li, Sara Jonsdottir Glaser, Rajni Hatti-Kaul

**Affiliations:** 1 Biotechnology and Applied Microbiology, Department of Process and Life Science Engineering, Kemicentrum, Lund University, Lund, SE-22100 Sweden; 2 Department of Botany and Microbiology, Faculty of Science, South Valley University, Qena, 83523 Egypt; 3 Present Address: Department of Chemistry, University of Copenhagen, Universitetsparken 5, Copenhagen, 2100 Denmark

**Keywords:** Furan Dicarboxylic acid (FDCA), *Gluconobacter oxydans*, Whole cell biocatalyst, Coniferyl aldehyde dehydrogenase (CALDH), Membrane-bound aldehyde dehydrogenase (MALDH), Biotransformation

## Abstract

**Background:**

2,5-Furandicarboxylic acid (FDCA) is a promising building block for biobased recyclable polymers and a platform for other potential biobased chemicals. The common route of its production is by oxidation of sugar-derived 5-hydroxymethylfurfural (HMF). Several reports on biocatalytic oxidation using whole microbial cells or enzymes have been reported, which offers potentially a greener alternative compared to the chemical process. HMF oxidases and aryl alcohol oxidases are the only enzymes able to catalyse the complete oxidation to FDCA, however at low concentrations and are subject to inhibition by the FFCA (5-formylfuran-2-carboxylic acid) intermediate. The present report presents a study on the oxidation of FFCA to FDCA using the obligately aerobic bacterium *Gluconobacter oxydans* and identification of the enzymes catalyzing the reaction.

**Results:**

Screening of three different strains showed *G. oxydans* DSM 50049 to possess the highest FFCA oxidation efficiency. Optimal reaction conditions for obtaining 100% conversion of 10 g/L (71 mM) FFCA to FDCA at 100% reaction yield were at pH 5, 30 °C and using 200 mg wwt /mL cells harvested at mild-exponential phase. In a reaction run at a 1 L scale using a total of 15 g/L (107 mM) FFCA supplied in a fed-batch mode, FDCA was obtained at a yield of 90% in 8.5 h. The product was recovered at 82% overall yield and 99% purity using a simple recovery process. Screening of several oxidoreductase enzymes from the gene sequences identified in the bacterial genome revealed two proteins annotated as membrane-bound aldehyde dehydrogenase (MALDH) and coniferyl aldehyde dehydrogenase (CALDH) to be the enzymes catalyzing the oxidization of FFCA.

**Conclusion:**

The study shows *G. oxydans* DSM 50049 and its enzymes to be promising biocatalysts for use in the FDCA production process from biomass. The high reaction rate and yield motivate further studies on characterization of the identified enzymes exhibiting the FFCA oxidizing activity, which can be used to construct an enzyme cascade together e.g. with HMF oxidase or aryl alcohol oxidase for one-pot production of FDCA from 5-HMF.

**Supplementary Information:**

The online version contains supplementary material available at 10.1186/s12934-025-02689-x.

## Background

The growing environmental concern for climate change and plastic pollution has motivated the development of fossil-free plastics that can be recycled or reused [1]. Mechanical recycling is the only mode of plastic recycling that is industrially practiced today and is limited to polyethylene, polypropylene, and polyethylene terephthalate (PET) bottles [2]. The latter, however, suffers from loss of molecular mass during recycling, which is attributed to its low glass transition temperature [3]. The introduction of rigid ring structures, e.g. aromatic or cycloaliphatic moieties into the polymer backbone has been shown to increase the glass transition temperature (T_g_) and thermal stability of PET-like polymers [4]. An alternative polymer that is potentially wholly biobased and that has captured industrial interest is poly(ethylene furanoate) (PEF) comprising ethylene glycol and 2,5-furan dicarboxylic acid (FDCA) [5].

The furan family of heterocyclic organic compounds with five-membered aromatic ring including furfural, 5-hydroxymethylfurfural (5-HMF), and their derivatives, is foreseen to comprise a useful source of biobased alternatives or substitutes to the chemicals and materials currently available [6, 7]. Besides serving as a monomer for recyclable polymers, FDCA is a potential platform chemical for several important chemicals such as succinic acid, 2,5-dihydroxymethylfuran, 2,5-dihydroxymethytetrahydrofuran, etc [8, 9].

**Scheme 1:**
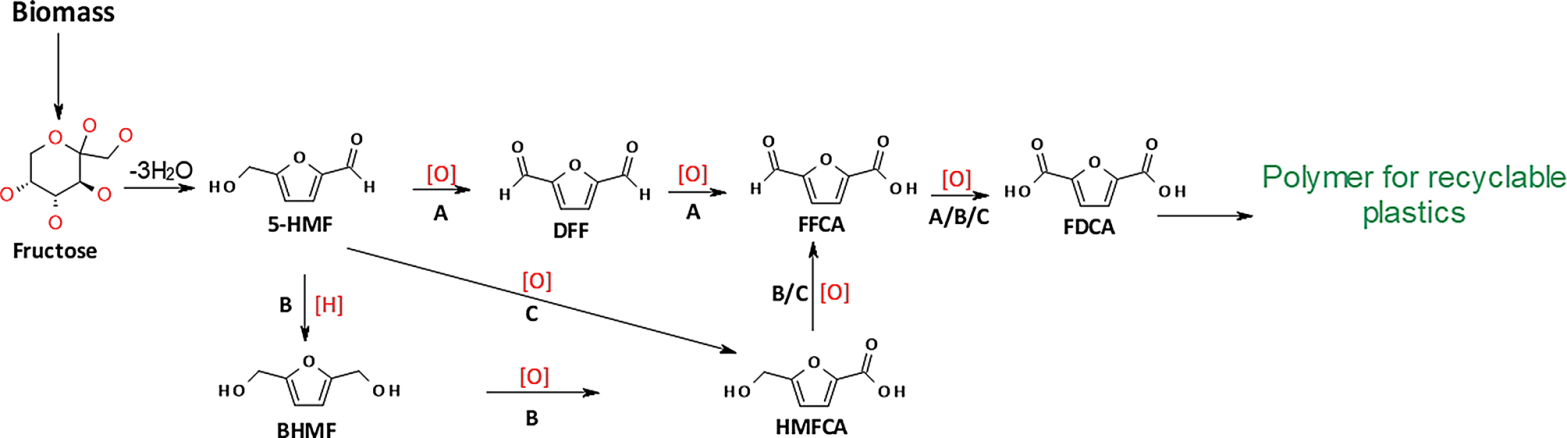
Possible pathways (**A**, **B** and **C**) to produce FDCA from 5-HMF obtained by dehydration of fructose. Pathway A involves oxidation of the hydroxyl group of 5-HMF to give DFF followed by stepwise oxidation of the aldehyde groups to form FFCA and then FDCA. In Pathway B, the aldehyde group on 5-HMF is first reduced to hydroxyl group to form BHMF, which is then oxidized via HMFCA to FFCA and finally to FDCA. Pathway C involves selective oxidation of the aldehyde group on 5-HMF to give HMFCA and the following oxidations are similar to that in Pathway B/C

Production of FDCA is normally achieved by the oxidation of 5-HMF, the dehydration product of C6 sugars [10, 11]. Oxidation of 5-HMF to FDCA needs three oxidation steps, which may be carried out using chemical, electrochemical, or biocatalytic processes (Scheme 1) [12–14]. Production of FDCA from 5-HMF via chemical catalysis is currently being scaled up but is based on expensive noble metal catalysts and requires energy demanding conditions [8, 10, 15]. Therefore, finding an alternative green process that can be performed under mild conditions using environment-friendly catalysts has attracted interest.

Whole-cell biocatalysts bearing enzymes with optimized function are used widely to produce different valuable chemicals that are building blocks for fine chemicals and pharmaceuticals [16, 17]. Within the last decade, many studies have reported the use of whole cells or enzymes to produce FDCA from HMF [18–20]. Most of the tested oxidative enzymes are limited to oxidation of either the aldehyde or the alcohol group, hence multi-enzymatic cascade reactions are required to achieve the production of FDCA [21–23], although giving low overall yield due to the difference in the optimum conditions of the different enzymes [15]. Until now, only HMF oxidases and aryl alcohol oxidases have shown the ability to catalyze the three oxidation steps, but the last step involving oxidation of FFCA to FDCA is a limiting step due to the inhibitory effect of FFCA at a concentration > 15 mM [24–27], hence requiring an additional enzyme for developing an efficient biocatalytic process for FDCA production from 5-HMF. Like its parent molecule 5-HMF, FFCA is a highly functional compound, possessing aldehyde and carboxylic groups, besides the furan ring, and constitutes a promising building block for polymers, fuels, chemical intermediates, and drugs [28]. Selective production of FFCA has also been shown by protecting the formyl group of 5-HMF and using an Au-catalyst [29]. FFCA was also the main product formed in our earlier studies on the oxidation of 5-HMF using the aryl alcohol oxidase from *Mycobacterium* sp [24]. Yet another route involving FFCA as an intermediate takes place via acid-catalyzed dehydration of 5-ketoaldonic acids formed from uronic acids found in abundance in agro-residues [30], and thus bypasses the unstable 5-HMF as the starting material.

*Gluconobacter oxydans*, an obligate aerobe, is known for its characteristic property of incomplete oxidation of sugars, alcohols, and aldehydes using a range of soluble and membrane-bound oxidases and dehydrogenases [31, 32]. The bacteria are used for large-scale production of several interesting compounds like ascorbic acid, sorbose, miglitol, etc. In our laboratory, we have shown the potential of the bacterium for selective oxidation of different primary alcohols and diols, such as 1-butanol and 1,6-hexanediol into the corresponding aldehydes and acids [33, 34]. In an earlier report, we have shown the use of *G. oxydans* cells for highly efficient oxidation of the formyl group of 5-HMF to form 5-hydroxymethyl-2-furancarboxylic acid (HMFCA), which is not modified further by the bacterium [35].

In the present study, we show the ability of *G. oxydans* cells to selectively and efficiently oxidize FFCA to FDCA under mild conditions and subsequently its recovery in pure form. Furthermore, the enzymes catalyzing the oxidation of FFCA were identified by genome screening for oxidoreductases, recombinant expression of the selected genes, and investigating their activity in vitro.

## Results and discussion

### Oxidation of FFCA to FDCA using *G. oxydans*

**Fig. 1:**
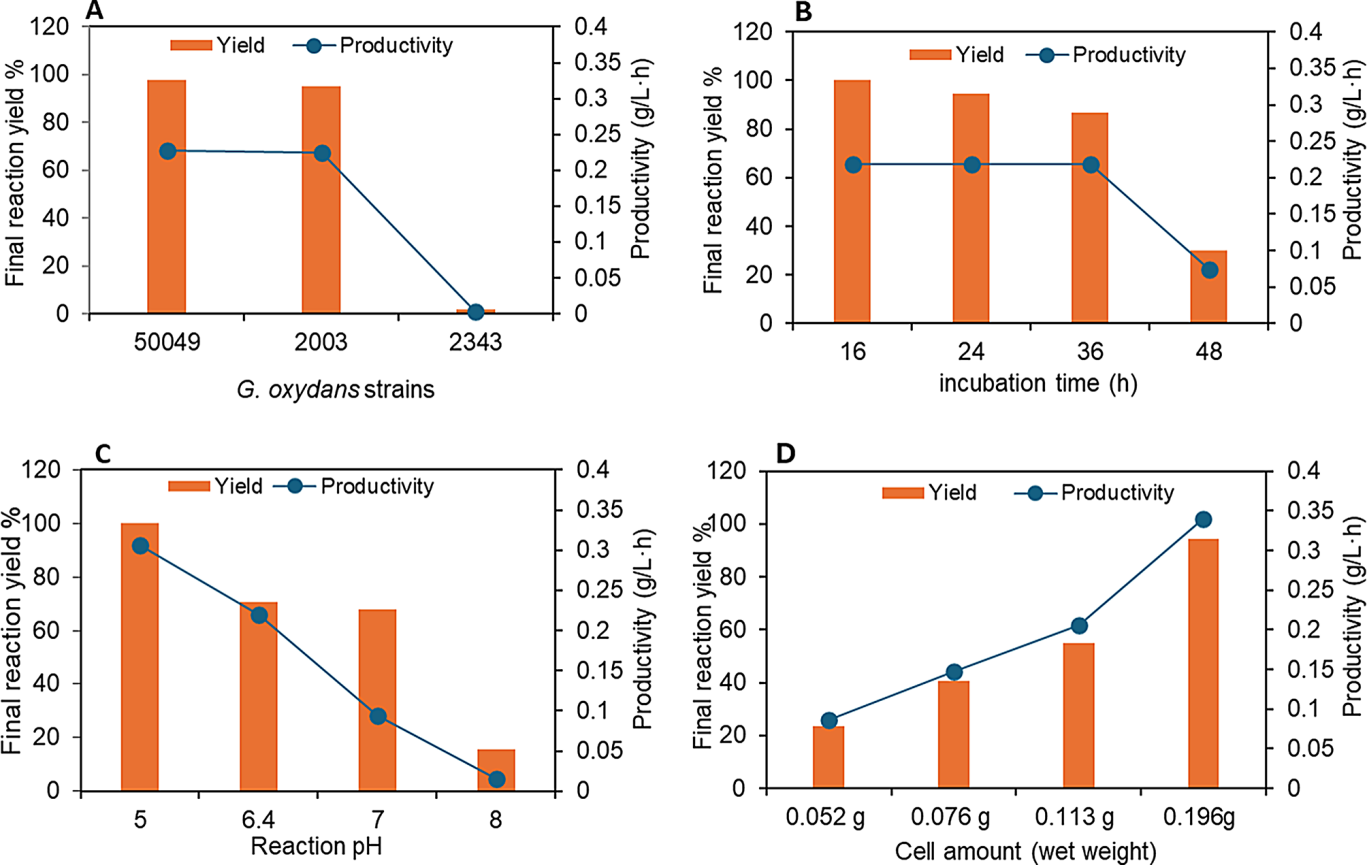
Optimization of different reaction parameters for oxidation of FFCA to FDCA catalyzed by resting *Gluconobacter oxydans* DSM 50049 cells in 0.1 M sodium acetate buffer pH 5 at 30 °C. The parameters included (**A**) *G. oxydans* strains (DSM 2003, DSM 2343, and DSM 50049), (**B**) cell cultivation time, (**C**) pH value, and (**D**) cell amount. The experiments for B, C and D were performed only with *G. oxydans* DSM 50049. The FFCA concentration used was 5 mg/mL in A, B and C, and 10 mg/mL in D

Initial experiments were performed to screen different *G. oxydans* strains, DSM 2003, 2343, and 50049, for their ability to oxidize FFCA to FDCA (Fig. 1A and S1A-C). The cells were grown in a glycerol medium that seems to have an activating effect on the expression of the sugar/polyol oxidizing enzymes [24]. The reaction against 5 mg/mL FFCA in 0.1 M acetate buffer pH 5 at 30 °C using the cells (collected from 4 mL culture and equivalent to 52 mg wet weight/mL of the reaction volume) showed that the strains 2003 and 50049 produced FDCA at a reaction yield > 90%, 100% selectivity and productivity of 0.2 g/L·h compared to *G. oxydans* DSM 2343 that gave only ~5% reaction yield (Fig. 1A and Figure S1). *G. oxydans* DSM 50049 cells were used for further investigations because not only was its activity slightly higher than that of DSM 2003 (Figure S1), but the strain also exhibited a shorter lag phase during cell cultivation as compared to the other strain. Screening of the optimal reaction parameters showed that *G. oxydans* DSM 50049 harvested at mid-exponential phase, i.e. 16 h of growth (Fig. 1B and S2), exhibited the highest activity in the reaction with 5 mg/mL FFCA at pH 5 to give FDCA with 100% reaction yield (Fig. 1C and S3).

Increasing the cell amount from 52 to 196 mg wwt/mL (corresponding to the culture volumes of 4 to 32 mL) and FFCA concentration to 8 mg/mL showed 100% conversion of FFCA, and > 80% yield of FDCA (Fig. 1D & S4). No other coproduct was observed; the lower reaction yield may be attributed to the enzyme inhibition and/or the lower aqueous solubility of FDCA [36]. The results obtained are in agreement with our earlier observations on the DSM 50049 strain oxidizing only the aldehyde group in 5-HMF to form HMFCA [35]. The organism does not exhibit any activity against the hydroxyl group on the furan ring.

**Fig. 2:**
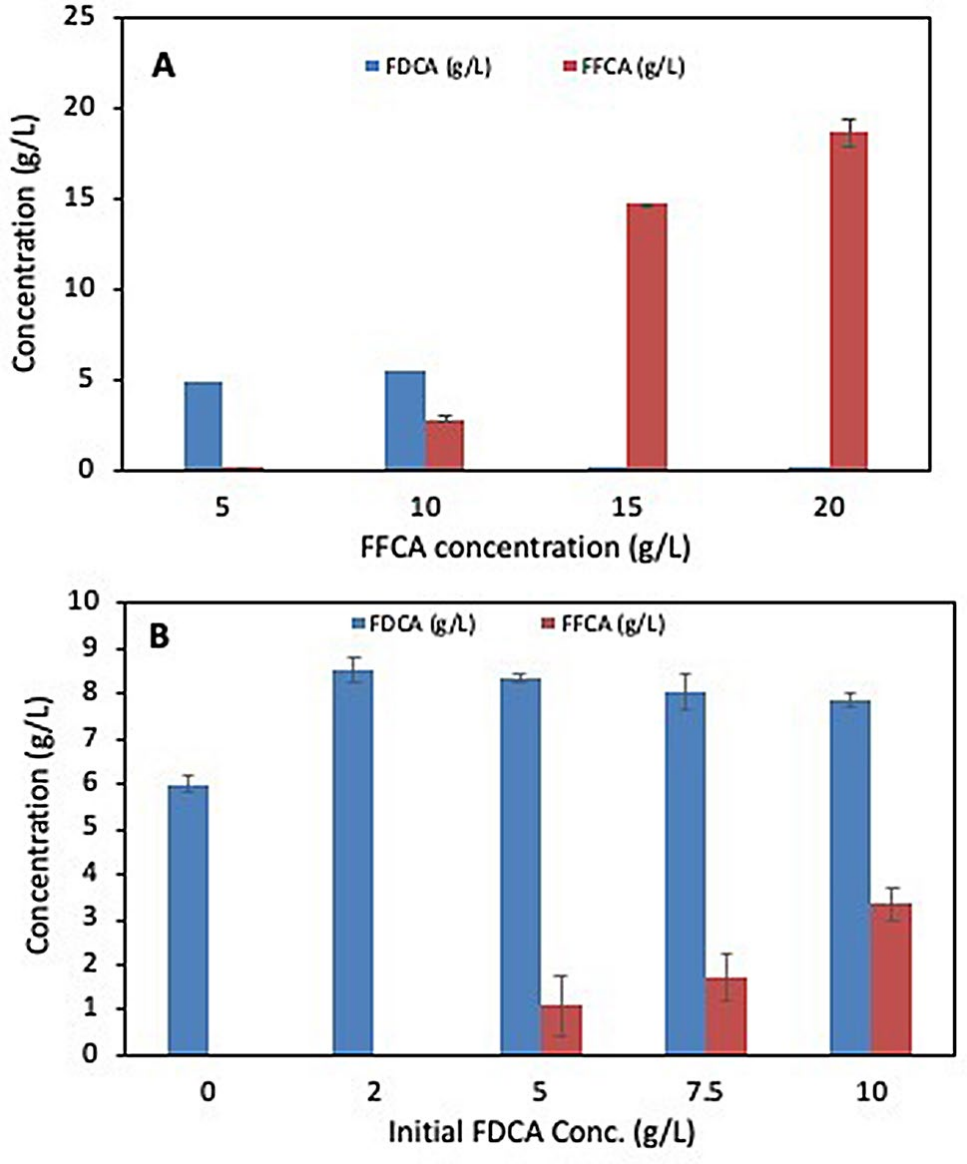
Effect of different concentrations of (**A**) FFCA and (**B**) FDCA on the oxidation activity of *G. oxydans* DSM 50049 against FFCA in 0.1 M acetate buffer pH 5 at 30 ºC. The amount of cells used for the reactions was 52 mg wet weight/mL, and in case of (**B**) the FFCA concentration used was 5 mg/mL

The inhibitory effect of FFCA on the catalytic activity of the *G. oxydans* cells was clearly observed at FFCA concentration of 10 mg/mL (at a cell concentration of 52 mg wwt/mL) when only 60% of the substrate was converted after 6 h of the reaction, and no more conversion was observed even with longer incubation up to 24 h (Fig. 2A and S5). No FDCA formation was noted with a further increase in the FFCA concentration to 15–20 mg/mL (Fig. 2A). According to earlier reports, FFCA concentration above 15 mM (2 mg/mL) has an inhibitory effect on fungal aryl alcohol oxidases (AAO) and HMF oxidase [15, 24]. Hence, *G. oxydans* and its enzymes seem to exhibit higher resistance to inhibition since FFCA could be used up to a concentration of 71 mM (10 mg/mL). Increasing the cell concentration for the reaction could overcome the inhibition to a certain extent (Fig. 1D).

The inhibitory effect of the product was also studied by including different concentrations of FDCA in the reaction with FFCA. It is important to note that FDCA has a limited solubility in aqueous solution (1.72 g/L at 313.5 K). Nevertheless, Fig. 2B and S6 show a slight inhibitory effect on the oxidative activity of *G. oxydans* DSM 50049 when the FDCA concentration was increased to 5 mg/mL, resulting in ~ 80% conversion of FFCA. Further increase in FFCA concentration to 7.5 and 10 mg/mL FDCA led to significant inhibition, and 32 and ~ 61% of FFCA remained unconverted, respectively, even after 24 h of reaction (Fig. 2B). This inhibition was ascribed to the decrease in pH of the reaction (to pH 4.3) due to the accumulation of FDCA. A similar observation was made by Wang et al. (2020) on inhibition of vanillin dehydrogenase activity used as a biocatalyst for 5-HMF oxidation to FDCA, and the inhibition was significantly reduced by neutralizing the pH [37, 38].

### Fed-batch biotransformation of FFCA to FDCA using *G. oxydans* DSM 50049

**Fig. 3:**
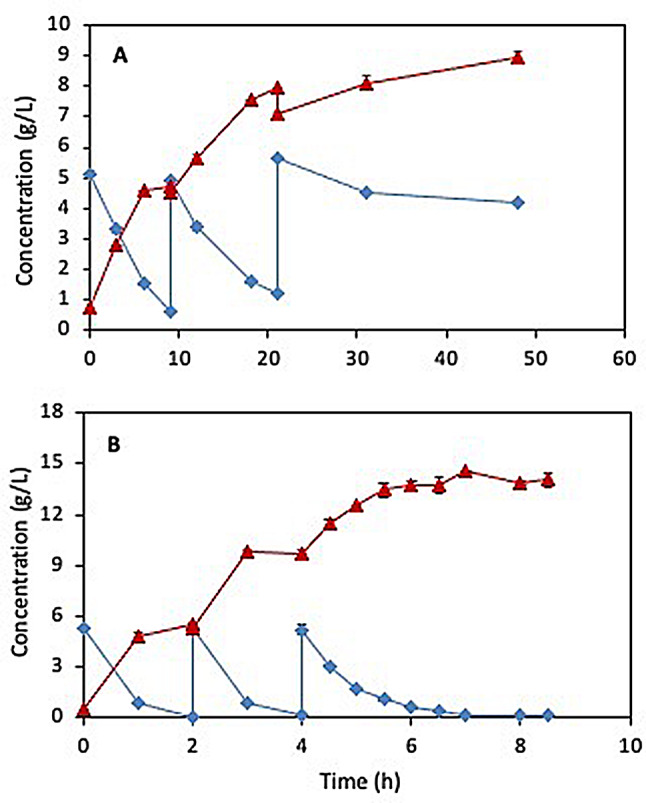
Fed-batch oxidation of FFCA (♦) to FDCA (▲) using resting *G. oxydans* DSM 50049 cells at 30 ºC in: (**A**) 50 mL 0.1 M acetate buffer pH 5 in 250 mL shake flask with uncontrolled pH and aeration, and (**B**) 1 L reaction volume in 3 L bioreactor maintained at pH 5 and 70% dissolved oxygen. The reaction was fed with FFCA stock solution to achieve the initial concentration of 5 mg/mL

To overcome the inhibitory effect of FFCA, fed-batch biotransformation was performed in a flask using a feed of 5 mg/mL FFCA in 50 mL acetate buffer pH 5 and without controlling pH and aeration. It took 9 h to achieve 90% FFCA conversion after the first feed and 12 h after the second feed when FDCA concentration reached around 8 g/L giving rise to increased acidity. A dramatic loss in cell activity was seen during the third feed, resulting in less than 10% FFCA conversion after 24 h (Fig. 3A). Only 8.1 g out of 15 g FFCA per liter added to the reaction, was converted to 9 g/L FDCA by the end of the experiment.

**Fig. 4:**
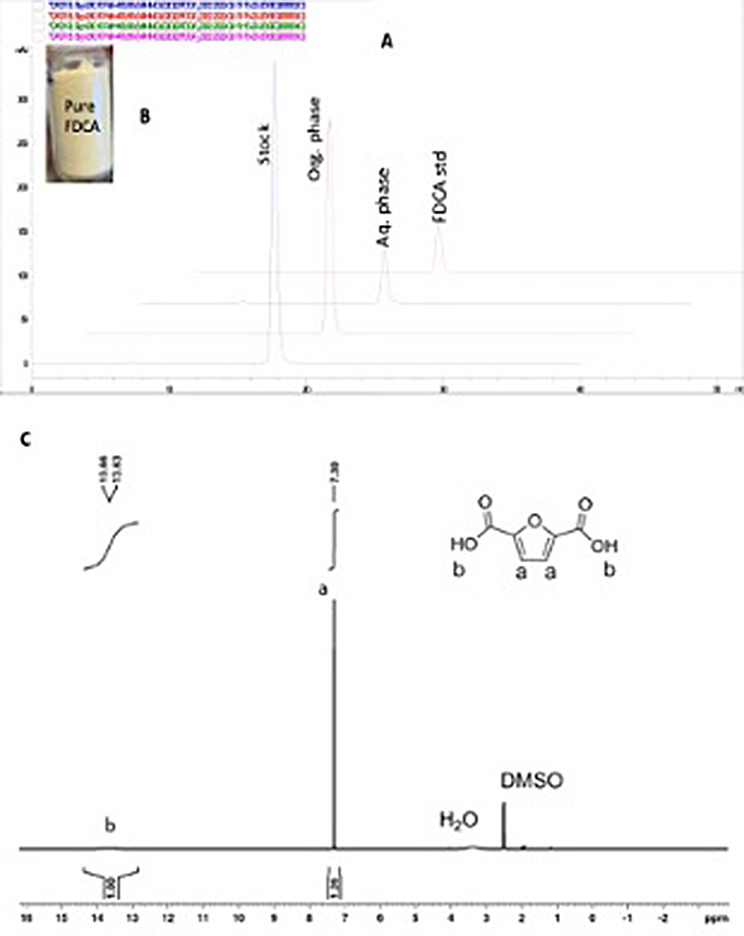
Analysis of the purification of FDCA product: (**A**) HPLC chromatograms of the final reaction solution obtained by *G. oxydans* DSM 50049 catalyzed oxidation of FFCA (1), FDCA recovered by extraction in ethyl acetate (2), FDCA remaining in the aqueous phase (3), and standard FDCA (4); (**B**) a picture of the purified FDCA, and (**C**) ^1^H-NMR of the purified FDCA, indicating > 99% purity

Subsequently, the fed-batch experiment was done in 1 L reaction volume with pH controlled at 5 and dissolved oxygen at 70%. As seen in Fig. 4B, FFCA in feeds 1 and 2 was converted efficiently to FDCA at 100% yield and selectivity within only 2 h, while it took 3 h for complete conversion of the last 5 g/L FFCA in the third feed (Fig. 3B). Overall, around 15 g/L FDCA with around 90% total reaction yield was obtained from 15 g/L FFCA within 8.5 h. Comparison of the formation of FDCA over time in the first batch shows that the initial activity of *G. oxydans* is 5-fold higher under controlled conditions of pH and aeration applied in the bioreactor compared to that under uncontrolled conditions (Fig. 3A and B) [37, 38]. The productivity during the first batch was 2.8 g/L·h compared to only 0.5 g/L·h FDCA obtained from the first batch in the shake flask. However, the drop in the overall productivity to 1.8 g/L·h in the controlled reactor and 0.2 g/L.h in the shake flask is due to the inhibitory effect of both product and substrate as described above, and the low solubility of FDCA could account to some extent for the observed drop in the reaction yield after the third feed. The decrease in activity may also be ascribed to insufficient regeneration of the cofactor required by the enzyme(s) catalyzing the reaction [39]. Hence, identifying the enzyme(s) involved in the oxidation process will provide valuable insights for optimizing biocatalyst and reactor design in future studies.

### Recovery and purification of FDCA

Since FFCA is converted selectively and nearly quantitively to FDCA, the product recovery and purification from the reaction solution at high purity was successfully achieved via a simple procedure involving first increasing the pH to 9 to ensure the solubilization of the product, centrifugation for separating the insoluble material including cells, filtering the supernatant to remove any remaining particulate matter before lowering the pH to 1.5. Around 80% of the FDCA was precipitated out from the solution, and > 70% of the remaining FDCA in the supernatant was recovered by liquid/liquid extraction (Table 1; Fig. 4). The final FDCA product was obtained with an overall yield of 82%, which was higher than that (76%) reported by Koopman et al. (2010) during the purification of FDCA from the reaction medium [40] and can be further improved to 87% by avoiding the washing step (Table 1). The purity of the recovered FDCA exceeded 99%, as verified by HPLC and proton NMR (Fig. 4A and C), indicating the high product quality for its use, especially in polymer synthesis.

**Table 1:** Purification of FDCA from the final reaction solution obtained from the fed-batch process of FFCA oxidation in 1 L reaction volume using the resting cells of *G. oxydans* DSM 50049. The solution contained 15 g/L of FDCA and no FFCA

Purification step	FDCA step yield (%)	FDCA overall yield (%)
Reaction solution	100	100
Cell removal	96.15	96.14
Concentration and precipitation	90.5	87
Washing and drying	94.3	82

### Identification of FFCA oxidizing enzyme(s)

The potential enzymes involved in the oxidation of FFCA to FDCA were screened from the *G. oxydans* DSM 50049 genome based on the conserved amino acid residues that are commonly present in the NAD(P)^+^, FAD, and PQQ-dependent oxidoreductases [40]. Over 100 genes encoding for oxidoreductases were identified, annotated, and classified based on the theoretical isoelectric point (pI) of the encoded protein and the cofactor identified for each annotated enzyme. Knowing from the literature that the *G. oxydans* whole cells carry out the oxidation reactions at pH in the acidic range and the cofactor dependence of enzymes catalyzing similar reactions, fourteen genes encoding oxidoreductases were selected (Table 2), amplified from the *G. oxydans* DSM 50049 genome and cloned into proper vectors. The nucleotide sequences of the selected genes and their corresponding amino acid sequences are shown in Figure S7. Seven constructs annotated as FAD-dependent oxidoreductase (39.9 kDa), alcohol dehydrogenase (ADH) (51.1 kDa), membrane-bound aldehyde dehydrogenase (MALDH) (83.1 kDa), xanthine dehydrogenase (XDH) (25.3 kDa), coniferyl aldehyde dehydrogenase (CALDH) (31.3 kDa), cyclohexadienyl dehydrogenase (CHDH) (24.1 kDa), and aldehyde oxidoreductase iron-sulfur binding (ALOD-Fe/SB) (54.8 kDa) were so far successfully cloned and transformed into different *E. coli* expression strains (BL21(DE3) and CodonPlus) for protein production (Table S3). Five of the seven proteins including MALDH, FAD-dependent oxidoreductase, ADH, CHDH, and ALOD-Fe/SB were successfully expressed in *E. coli* BL21 (DE3) grown in LB medium at 16 °C and induction with 0.5 mM IPTG. The expression of the other two proteins, i.e. xanthine dehydrogenase and coniferyl aldehyde dehydrogenase (CALDH) was only possible in *E. coli* CodonPlus grown in LB (and induced with 0.5 mM IPTG) and in autoinduction medium, respectively (Table S3, Figure S8A-C). Screening of enzyme activities using the whole recombinant *E. coli* cells showed only two sets of cells - expressing the enzymes annotated as MALDH and CALDH, respectively – to display activity against FFCA to form FDCA.

**Table 2:** Fourteen genes are selected from *G. oxydans* based on pI values, and their conserved amino acid residues. Names, annotations and expected sizes are indicat

No.	Gene Name	Annotation	Theoretical pI	Anticipated cofactor	Size (bp)
1	ddmA1	FAD dependent oxidoreductase	5.1	FAD	887
2	adhB2	Alcohol dehydrogenase		NAD^+^	1461
3	GO50049LU-2_1_00206	GMC family oxidoreductase	8.5	FAD	1227
4	GO50049LU-2_1_02677	Membrane-bound aldehyde dehydrogenase	8.8	FAD molybdopterin guanine dinucleotide [4Fe-4 S] cluster	2334
5-1	sldA_1	Glycerol dehydrogenase large subunit	6.6	PQQ	321
5-2	sldA_2				1659
6	ctcP	FAD-dependent oxidoreductase	8.5	FAD	903
7	rfbD	FAD-dependent oxidoreductase	6.2	FAD	1164
8	GO50049LU-2_1_02764	Xanthine dehydrogenase	5.1	NAD^+^	461
9	GO50049LU-2_1_02763				294
10	GO50049LU-2_1_01181	GMP reductase	4.1	NAD(P)^+^	377
11	adhA1	Alcohol dehydrogenase	6.6	NAD^+^	750
12	calB	Coniferyl aldehyde dehydrogenase	9.6	FAD	879
13	GO50049LU-2_1_01307	HMF oxidase	8.8	FAD	2072
14	tyrC	Cyclohexadienyl dehydrogenase	5.0	NAD^+^	678
15	paoA	Aldehyde oxidoreductase iron-sulfur-binding	6.9	FAD [4Fe-4 S] cluster	531
16	GO50049LU-2_1_00575				116
17	paoB				935

The membrane-bound aldehyde dehydrogenases are mostly PQQ-dependent enzymes [41]. The *G. oxydans* DSM 50049 MALDH has the conserved amino acid residues (GxGxxG) common for flavoproteins and was identified with 66.6% identity to an uncharacterized pyrroloquinoline quinone dependent MALDH (using non-redundant UniProtKB/SwissProt sequences database) (Figure S9A) [42]. However, when the NCBI Protein Reference Sequences database was used, a molybdopterin-dependent oxidoreductase was identified with 100% identity [43]. The latter enzyme is a dimer of heterotrimer comprising 88.7 kDa molybdoprotein large subunit, 30.2 kDa flavoprotein medium subunit, and 17.8 kDa iron-sulphur domain small subunit [44]. On the other hand, *G. oxydans* DSM 50049 MALDH comprised only 83.1 kDa protein domain. Experimentally, the *E. coli* cells with overexpressed MALDH showed activity in the presence of FAD, and the addition of sodium molybdate to the culture during protein expression enhanced the enzyme activity against FFCA (data not shown). This result fits with the blast search and conserved motif indicating that the enzyme is molybdopterin-dependent rather than PQQ-dependent.

The CALDH enzymes are NAD^+^-dependent oxidases that oxidize coniferyl aldehyde to ferulic acid [45]. The closest related enzyme to CALDH is a coniferyl aldehyde dehydrogenase from *Pseudomonas* sp. HR199 (sequence ID: O86447.3) [45] with a sequence coverage of 95% and sequence identity of 46.3% (Figure S9B).

Interestingly, both MALDH and CALDH align, although with only 25% and 28% sequence identity, with the molybdenum-dependent periplasmic oxidoreductase and a cytoplasmic dehydrogenase enzyme, respectively, in *Pseudomonas taiwanensis* VLB120 and *Pseudomonas putida* KT2440 that were identified recently by extended gene deletions to be involved in oxidative detoxification of HMF [46]. The pterin cytosine dinucleotide (MCN) and dioxothiomolybdenum (VI) (MOS) binding sites were found to be conserved in MALDH and the periplasmic oxidoreductase as in the crystal structure of an molybdoenzyme (aldehyde oxidase) from *E. coli* (PDB:5G5G) (Figure S10) [47]. Similarly, the active site residue and NAD^+^ binding site in CALDH and the *Pseudomonas* cytoplasmic dehydrogenase were conserved and matched those in the crystal structure of a related aldehyde dehydrogenase from *Bacillus cereus* (PDB: 5GTK) (Figure S11). This provides additional evidence of the role and cofactor dependence of the enzymes identified from *G. oxydans* DSM 50049.

**Fig. 5:**
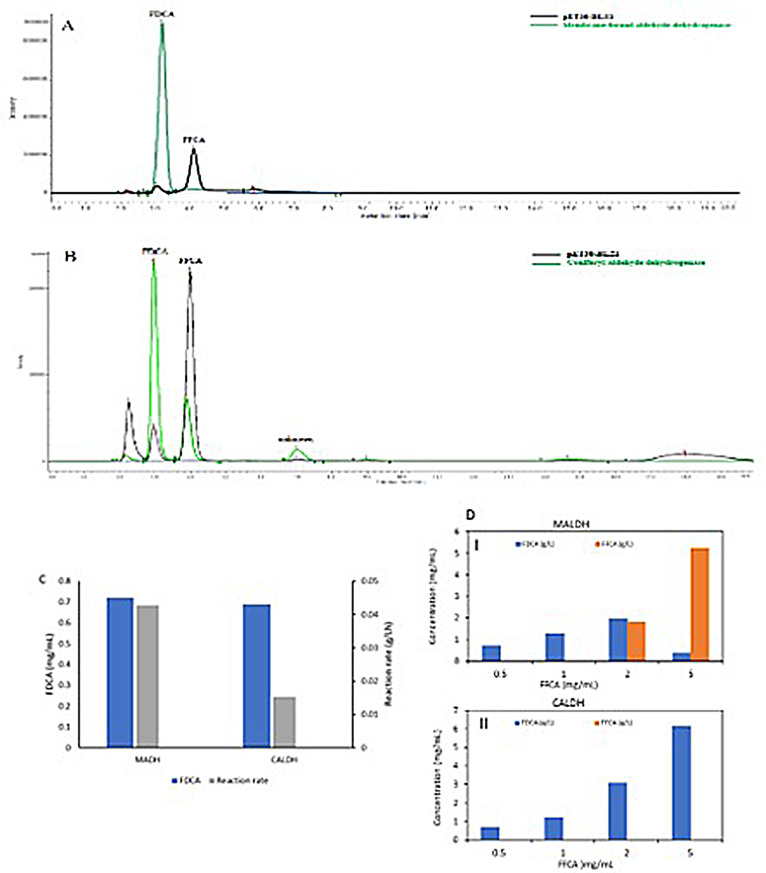
Oxidation of FFCA to FDCA using recombinant *E. coli* (lysate and whole cells) bearing *G. oxydans* DSM 50049 membrane-bound aldehyde dehydrogenase (MALDH) and coniferyl aldehyde dehydrogenase (CALDH), respectively. (**A**) HPLC analysis of samples from the reaction catalysed by the insoluble fraction of the cells expressing MALDH (green chromatogram), in comparison with the reaction using negative control *E. coli* lysate (black chromatogram), (**B**) HPLC analysis of samples from the reaction performed with the soluble fraction of CALDH containing cells (green chromatogram), in comparison with control reaction using negative control *E. coli* lysate (black chromatogram), (**C**) FDCA concentration after 24 h reactions catalysed by 1.94 mg cell dry weight/mL of the whole cells overexpressing MALDH and CALDH, respectively, against 0.5 mg/mL FFCA and the corresponding initial reaction rates, (**D**) Activity of MALDH (DI) and CALDH (DII) against different concentrations of 0.5, 1, 2 and 5 g/L of FFCA

The recombinant *E. coli* cells expressing MALDH exhibited activity against FFCA, however, on cell lysis, only the insoluble fraction showed activity (Fig. 5A, C). This observation may confirm that MALDH is a membrane bound enzyme that includes a signal peptide (data not shown), and hence the activity is located in the insoluble fraction (containing membrane) of the cell lysate. On the other hand, the CALDH activity resided in the soluble fraction of the cell lysate (Fig. 5B, C). Activity measurements using the same concentration of the recombinant *E. coli* expressing the two enzymes showed that MALDH-containing cells exhibited 2.7 times higher activity against 0.5 mg/mL FFCA compared to CALDH (Fig. 5C). This could partly be due to a possible higher level of MALDH expression, however, CALDH exhibited significantly higher activity when FFCA concentration was increased to 5 mg/mL, giving 100% conversion of FFCA to FDCA compared to only 6% conversion with MALDH (Fig. 5DI and II). The enzymes need to be investigated in more detail to provide deeper insight into the enzyme structure, function, and their roles in the oxidation of furans.

## Conclusions

Through this study, we show *G. oxydans* DSM 50049 to be a promising source of oxidative enzymes for furan transformations under mild conditions. The wild-type bacteria could be used directly for selective and efficient production of FDCA from FFCA, and the enzymes involved in the oxidation were identified. We noted that the FFCA oxidation activity was not common to all the *G. oxydans* strains under the conditions used. Although the biocatalytic activity was sensitive to inhibition by FFCA, FDCA, and low pH, fed-batch bioconversion with pH control and sufficient aeration provided a sufficiently efficient process for FDCA production. Further enhancement can be achieved by improving the tolerance of the bacterium to the furan derivatives, incorporating a cofactor regeneration system, and designing the process to enable in situ product removal. The low aqueous solubility of FDCA, particularly at low pH, provided a simple route for its recovery at high overall yield and purity. The wild-type *G. oxydans* DSM 50049 lacks the enzyme(s) able to catalyze oxidation of the hydroxyl group linked to the furan ring and hence does not allow the production of FDCA directly from 5-HMF, which is oxidized instead to HMFCA and not further to any other oxidation product [35]. The identified enzymes in *G. oxydans* DSM 50049 exhibiting the FFCA oxidizing activity will enable the construction of an enzyme cascade together e.g. with HMF oxidase or aryl alcohol oxidase for one-pot production of FDCA from 5-HMF in a heterologous host [24, 27]. Alternatively, FDCA production by the *G. oxydans* can be achieved by engineering the cells with a gene encoding the enzyme oxidizing the hydroxyl group in HMFCA, e.g. HMF/furfural oxidoreductase (hmfH) (to be reported in another study) [48].

## Materials and methods

### Materials

*G. oxydans* strains DSM 2343, 2003, and 50049 were obtained from the DSMZ culture collection (Braunschweig, Germany). FFCA was procured from AA BLOCKS (San Diego, CA, USA), and FDCA was from Sigma, while other chemicals were purchased from Merck. All chemicals were of analytical grade.

### FFCA oxidation using *G. oxydans*

Lyophilized cells of *G. oxydans* DSM 50049, 2003, and 2343 were inoculated into 50 mL *Gluconobacter* broth medium in 250 mL flasks, containing per liter: 100 g glucose and 10 g yeast extract at pH 6.8. The flasks were incubated in a shaker incubator (Ecotron, Infors HT, UK) at 30 °C and 200 rpm for 24 h. The bacterial culture was then stored in 1 mL aliquots of 20% (v/v) glycerol at -20 °C for further use.

For preparing the activated cells for FFCA oxidation, 100 µL glycerol stock of *G. oxydans* was inoculated into 50 mL medium in a 250 mL flask, containing per liter: 25 g glycerol and 10 g yeast extract with pH adjusted to 5 and incubated as described above. Thereafter, the culture broth was centrifuged at 4700 xg for 15 min (Sorvall LYNX 4000, Thermo Scientific, Germany), and the cell pellet was separated and washed twice using 0.1 M sodium phosphate buffer pH 7 before use in the oxidation reactions.

The effect of various reaction parameters on the oxidation activity of the cells was studied. For screening of different species, cell pellets of *G. oxydans* DSM 50049, 2003, 2343 of the activated strains were collected from 4 mL cultivation medium (OD around 2) and re-suspended in 1 mL of 100 mM sodium acetate buffer pH 5 in 4 mL reaction vials. All vials were supplemented with 5 mg/mL FFCA, and incubated in a thermomixer (MKR 13, HLC Biotech, Germany) at 30 °C and 500 rpm without pH control. Fifty microliter samples were collected during the reaction to analyze substrate and product concentrations. All experiments were carried out in duplicates.

Reaction parameters including pH, cultivation time of the cells, cell concentration, FFCA, and FDCA concentration (for substrate and product inhibition) were evaluated using the activated *G. oxydans* DSM50049 cells. For testing the effect of the pH, the cell pellet from 4 mL culture broth was re-suspended in 1 mL of 100 mM buffers, sodium acetate pH 5, or sodium phosphate pH 6, 7, and 8. The effect of the cultivation time (growth phase) was tested by collecting the cells during cultivation at 16, 24, 36, and 48 h and testing the activity against 5 mg/mL FFCA in 0.1 M acetate buffer pH 5. The effect of different concentrations of FFCA (5, 10, 15, and 20 g/L) and of FDCA (2, 5, 7, 10 g/L), respectively, on the cell activity was studied in 0.1 M acetate buffer pH 5 using the cell pellets collected from 4 mL culture broth. The effect of cell amount was examined by mixing 52, 76, 113, and 196 mg wet weight (wwt), respectively, with 1 mL of the sodium acetate buffer pH 5 supplemented with 10 mg/mL FFCA. Samples of 50 µL volume were collected every 0, 3, 6, 9, 12, 24, and 48 h for analyses of the residual substrate and the product formed.

### Oxidation of FFCA to FDCA using *G. oxydans* cells in fed-batch mode

Oxidation of FFCA using *G. oxydans* DSM 50049 cells in fed-batch mode was carried out in different volumes of 0.1 M acetate buffer pH 5. For the reaction in a 50 mL scale, the cells collected from 200 mL culture were washed and resuspended in the buffer supplemented with 5 mg/mL FFCA in a 250 mL flask. The reaction was started by incubation at 30 °C in a shaker incubator with a shaking speed of 200 rpm. The conversion profiles were followed by the analysis of the samples collected every 3 h. The second feed of 5 mg/mL was added (by feeding 1 mL of 50 mg/mL FFCA stock solution) after 9 h of the reaction, at which time > 90% of the substrate had undergone conversion. The same procedure was repeated for the third feed.

For the experiment in 1 L scale, *G. oxydans* DSM 50049 cells from 4 L culture were harvested by centrifugation at 8500 ×g and 4 °C for 20 min (Sorvall, Thermo Scientific, Germany), the cell pellet washed once with 500 mL of 0.1 M acetate buffer pH 5, recentrifuged, and resuspended in the buffer supplemented with 5 mg/mL FFCA in a 3 L bioreactor (Applikon, Microbial Biobundle, The Netherlands). The biotransformation was performed at 30 °C and pH was controlled at 5 by the addition of 5 N NaOH. Dissolved oxygen (DO) was maintained at 70% through controlling the stirrer speed. Two 50 mL feeds of 100 g/L FFCA were added to the bioreactor at 2 h and 4 h of the reaction. Seven-milliliter samples were collected every hour, of which 5 mL was discarded and 2 mL was used for analysis of the substrate and the product.

### Recovery and purification of FDCA

The pH of FDCA solution from the one-liter fed-batch experiment was first increased to pH 9.0 with 5 M NaOH, followed by centrifugation at 4500 rpm, 4 °C for 20 min (Sigma 3-16PK, Sigma, Germany). The cell pellet was discarded, and the liquid was heated in a water bath at 90 °C for 10–15 min, after which the sediment was removed by centrifugation at 8500 ×g and 4 °C for 20 min. This was followed by vacuum filtration using a 0.45 μm filter. The pH of the filtrate was then adjusted to pH 1 using concentrated HCl (36%) (Alfa Aesar). Thereafter, the precipitated FDCA was collected by centrifugation and the residual water was evaporated by placing the product sample in the oven at 50 °C. The FDCA remaining in the supernatant was recovered by liquid-liquid extraction with an equal volume of ethyl acetate and obtained in a concentrated form by rotary evaporation (Rotavapor R-300, Büchi, Germany). The residual ethyl acetate in the concentrated FDCA was removed by subjecting the sample to vacuum overnight. The purity of FDCA was confirmed by analysis using HPLC and NMR.

### Screening of *G. oxydans* DSM 50049 genome for the genes encoding enzymes responsible for FFCA oxidation

The whole genome of *G. oxydans* DSM 50049 was screened for potential oxidoreductases using SnapGene (Dotmatics, Boston, USA). The screening was based on the conserved binding motifs, GxGxxA, GxGxxG, xRNxV, xRNxQ, identified for the NAD(P)^+^, FAD, and PQQ cofactors, respectively, utilized by these groups of enzymes [49]. For further analysis of the identified genes, the sequences were blasted on NCBI Blast+, Uniprot Blast, and Expasy BlastP online tools [50]. The physical and chemical properties of the identified proteins were calculated using the Expasy ProtParam tool [51]. The selection of the hits to be further studied was based on the theoretical isoelectric point (pI). Sequence alignment was made using clustal-Omega (1.2.4) program from ClustalW [52] and the active site residues, conserved domains, and cofactor binding sites were determined by comparing to characterized enzymes from Protein Data Bank (PDB:5G5G and GTK). The alignment was generated using Jalview software and the colouring of the resides was based on sequence identity percentage.

### Protein production and screening for enzyme activity against FFCA

Fourteen genes encoding oxidoreductases (Table S4) with probable activity against the target substrate (FFCA) were amplified using Phusion™ High–Fidelity DNA Polymerase (Thermo Fisher Scientific) from the isolated genome of *G. oxydans* DSM 50049 via designed primers with proper restriction sites, following the manufacturer´s protocol (Table S1). The amplified genes were purified by GeneJET PCR purification kit (Thermo Scientific, USA) or QIAquick^®^ gel extraction kit (Qiagen, Germany). Finally, the DNA fragments were cloned into a plasmid vector, either pET-30a (+) through double digestion (Fast digest restriction enzymes NdeI, HindIII or XhoI, Themo Scientific, USA) and ligation (T4 DNA ligase, NEB, Massachusetts, USA), or pET-28a (+) (Novagen, Madison, WI, USA) using Gibson assembly for multi-fragment genes following the manufacturer´s protocol (NEBuilder^®^ HiFi DNA Assembly Master Mix, New England Biolabs). Table S2 summarizes the conditions used for cloning of the target genes.

The cloned vectors were transformed into *E. coli* DH5α competent cells (Thermo scientific) following the protocol provided by the manufacturer (Cat. Number 18265-017). The recovered cells were screened for the correct assembly via colony PCR (DreamTaq Green PCR Master Mix (2X), Themo Scientific, USA) and Sanger sequencing (Eurofins Genomics, Germany GmbH) for further confirmation. The correct clones were transformed into expression host *E. coli* strains (BL21(DE3) and BL21 CodonPlus).

Protein production was conducted in LB medium supplemented with 50 µg/mL kanamycin (for both pET28 and pET30 clones) initially at 37 °C, 200 rpm, and the gene expression was initiated at OD_600nm_ of 0.6–0.8 using 0.5 mM IPTG (isopropyl β-D-1-thiogalactopyranoside). The induced cultures were then transferred to 16–18 °C for 20 h before harvesting by centrifugation at 9700 ×g for 30 min. The cell pellets were resuspended in 100 mM Tris-HCl, 0.5 M NaCl, pH 8.0 or 0.1 M acetate buffer pH 5, followed by cell lysis by sonication (Branson Sonifier 250) in pulses of 20–50 kHz for 2 min each in an ice bath. Finally, the cell lysate was separated into soluble and insoluble fractions by centrifugation (Thermo Scientific™ Sorvall LYNX 6000 Superspeed Centrifuge, USA) at 27 000 ×g for 30 min at 4 °C. The protein content in both fractions was analyzed using SDS-PAGE (12% Mini-PROTEIN TGX Stain-Free Gel, Bio-Rad). For improving the production and solubility of the enzymes under investigation, different conditions were examined, including the cultivation medium (LB, autoinduction), IPTG concentration (0.1-1 mM), growth temperature (16–30 °C), and length of incubation after induction (4 and 24 h). Table S3 summarizes the tested and final conditions to produce the target enzymes.

### Determination of enzyme activity

The activity of the expressed enzymes was determined in 1 mL reaction volume in 96 deep well plate using 200 µL of enzyme samples (whole cells, soluble and insoluble fractions), 5 mM substrate (FFCA), 1–5 mM cofactor (NAD(P)^+^, FAD), at a shaking speed of 800 rpm (Eppendorf plate shaker) and 30 °C. Samples of 50 µL were taken at 3, 5 and 24 h for analysis.

### Kinetics parameters

The reaction yield (Y), volumetric productivity (Q_P_), and substrate conversion (%) were calculated using the following equations:

$$\begin{gathered}\:Y\:(mole\:product\:/mole\:substrate)\:\left( \% \right)\: \hfill \\= \:(({P_{final}}\left( {mM} \right))/{S_{initial}}\:(mM))*100 \hfill \\ \end{gathered} $$

$$\:{Q}_{P}\:(g/L.h)\:=\:(P\hspace{0.17em}-\hspace{0.17em}{P}_{0})/(t\hspace{0.17em}-\hspace{0.17em}{t}_{0})$$

$$\:Conversion\:\left(\%\right)\:=\:(1\hspace{0.17em}-\hspace{0.17em}S/{S}_{0})\:\times\:\:100$$

where *P* is the product concentration, *P*_*0*_ initial product concentration, *S* and *S*_*0*_ are the final and initial substrate concentrations, respectively, *t* is the reaction time, while *t*_0_ is the starting point of the reaction.

### Analytical procedures

The concentrations of FFCA and FDCA were determined using HPLC (JASCO, Tokyo, Japan) equipped with Fast Acid Analysis HPLC Column connected to a guard column (BioRad, Sweden), Jasco refractive index detector, a JASCO UV detector operating at 254 nm, and a JASCO intelligent autosampler. The column temperature was maintained at 65 °C in a JASCO oven. Samples were diluted with 40% DMSO in water and then filtered using 0.45 μm filters. A 10 µL aliquot was injected in 0.5 mM sulfuric acid mobile phase at a flow rate of 0.6 mL/ min. The peaks for the different compounds were confirmed and quantified using external standards. The purity of FDCA was further confirmed by proton NMR (DMSO-*d6*) using 400 MHz NMR (Bruker, UltraShield Plus 400, Germany).

## Electronic supplementary material

Below is the link to the electronic supplementary material.

Supplementary Material 1

## Data Availability

No datasets were generated or analysed during the current study.
