# Supplementary material for: Gluconobacter oxydans DSM 50049 – an efficient biocatalyst for oxidation of 5-formyl-2-furancarboxylic acid (FFCA) to 2,5-furandicarboxylic acid (FDCA)
Source: Microb Cell Fact. 2025 Mar 19;24:68. doi: 10.1186/s12934-025-02689-x (PMC11924602; doi:10.1186/s12934-025-02689-x)
Supplement: Supplementary file 1 — Supplementary Material 1 [file 12934_2025_2689_MOESM1_ESM.docx]

**Supporting information**

*Gluconobacter oxydans* DSM 50049 *–* an efficient biocatalyst for oxidation of 5-formyl-2-furancarboxylic acid (FFCA) to 2,5-furandicarboxylic acid (FDCA)

Mahmoud Sayed^1,2*^, Mohamed Ismail^1^, Anirudh Sivasubramanian,

Riko Kawane, Chengsi Li, Sara Jonsdottir Glaser^♠^ and Rajni Hatti-Kaul ^1*^

^1^ Biotechnology, Department of Chemistry, Center for Chemistry and Chemical Engineering, Lund University, SE-22100 Lund, Sweden

^2^ Department of Botany and Microbiology, Faculty of Science, South Valley University, 83523 Qena, Egypt

^♠^ Department of Chemistry, Copenhagen University, 52100 Copenhagen, Denmark

** Corresponding authors*

E-mails: [mahmoud.sayed_ali_sayed@biotek.lu.se](mailto:mahmoud.sayed_ali_sayed@biotek.lu.se); rajni.hatti-kaul@biotek.lu.se

**Figure S1.** Screening of different *G. oxydans* strains including (A) DSM 50049, (B) DSM 2003, and (C) DSM 2343 for the oxidation of 5 mg/mL FFCA (♦) to FDCA (▲) in 0.1M acetate buffer pH 5 at 30 °C and 200 rpm. The reaction was conducted in 1 mL volume and cells were obtained from 4 mL culture broth (52 mg wet weight cells) of the *G. oxydans* strains.

**
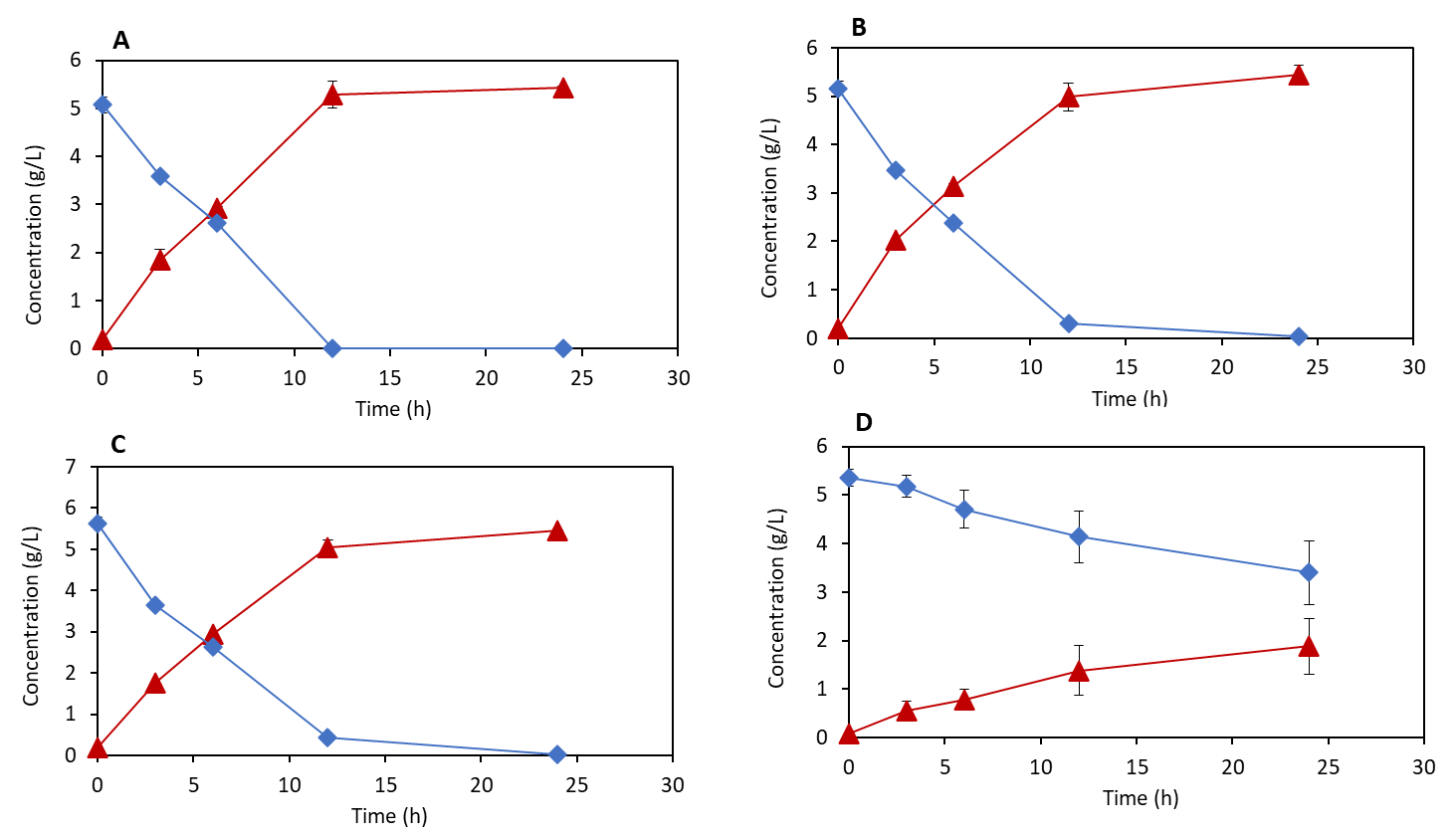
**

**Figure S2**. Oxidation of 5 mg/mL FFCA (♦) to FDCA (▲) in 0.1M acetate buffer pH 5 at 30 ⁰C using resting cells of *G. oxydans* DSM 50049 cultivated for (A)16 h, (B) 24 h, (C) 36 h, and (D) 48 h, respectively.

**Figure S3**. Oxidation of 7 mg/mL FFCA (♦) to FDCA (▲) using resting cells of *G. oxydans* DSM 50049 in (A) 0.1M acetate buffer pH 5, (B) 0.1M phosphate buffer pH 6.4, (C) 0.1M phosphate buffer pH 7, and (D) 0.1M phosphate buffer pH 8 at 30 °C.

**Figure S4.** Oxidation of 8 mg/mL FFCA (♦) to FDCA (▲) using different concentrations of resting cells of *G. oxydans* DSM 50049: (A) 52 mg wet weight/mL, (B) 76 mg wwt/mL, (C) 113 mg wwt/mL, and (D)196 mg wwt/mL, in 0.1M acetate buffer of pH 5 at 30 °C.

**
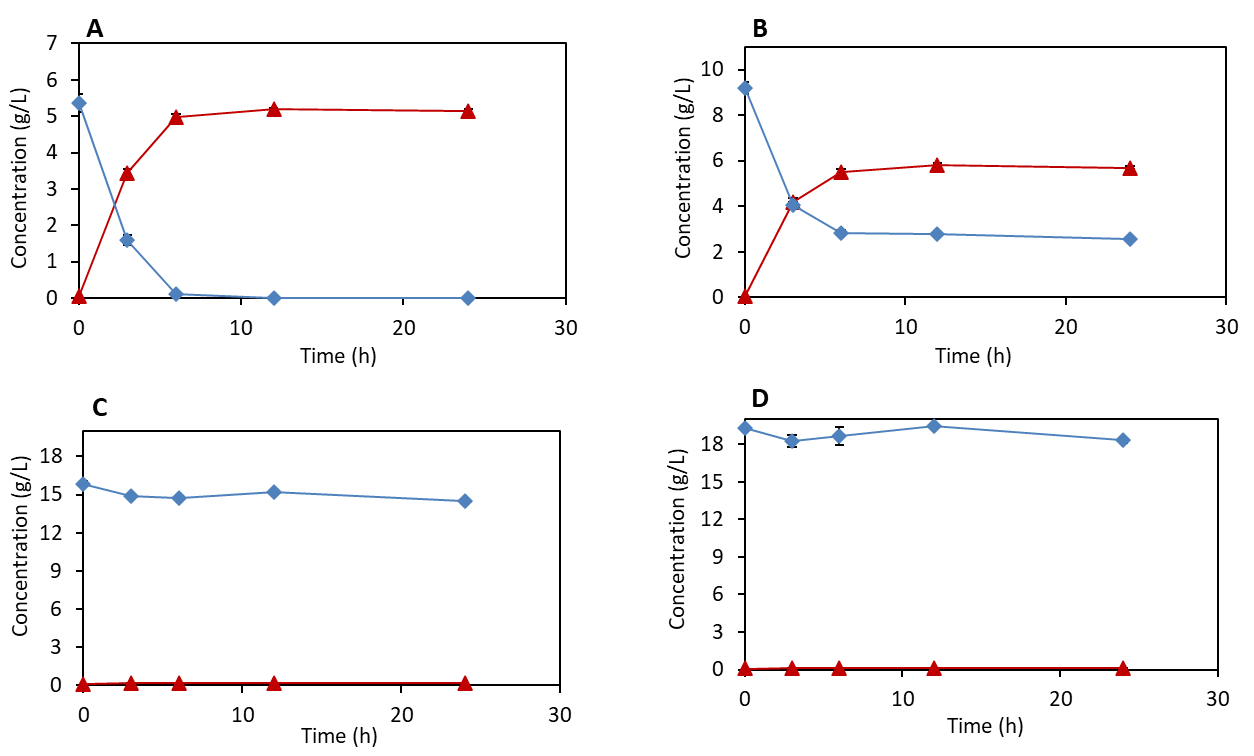
**

**Figure S5**. Effect of different FFCA concentrations: (A) 5 mg/mL, (B) 10 mg/mL, (C) 15 mg/mL, and (D) 20 mg/mL on the oxidation of FFCA (♦) to FDCA (▲) using 52 mg wwt/ml resting cells of *G. oxydans* DSM 50049 in 0.1M acetate buffer pH 5 at 30 °C.


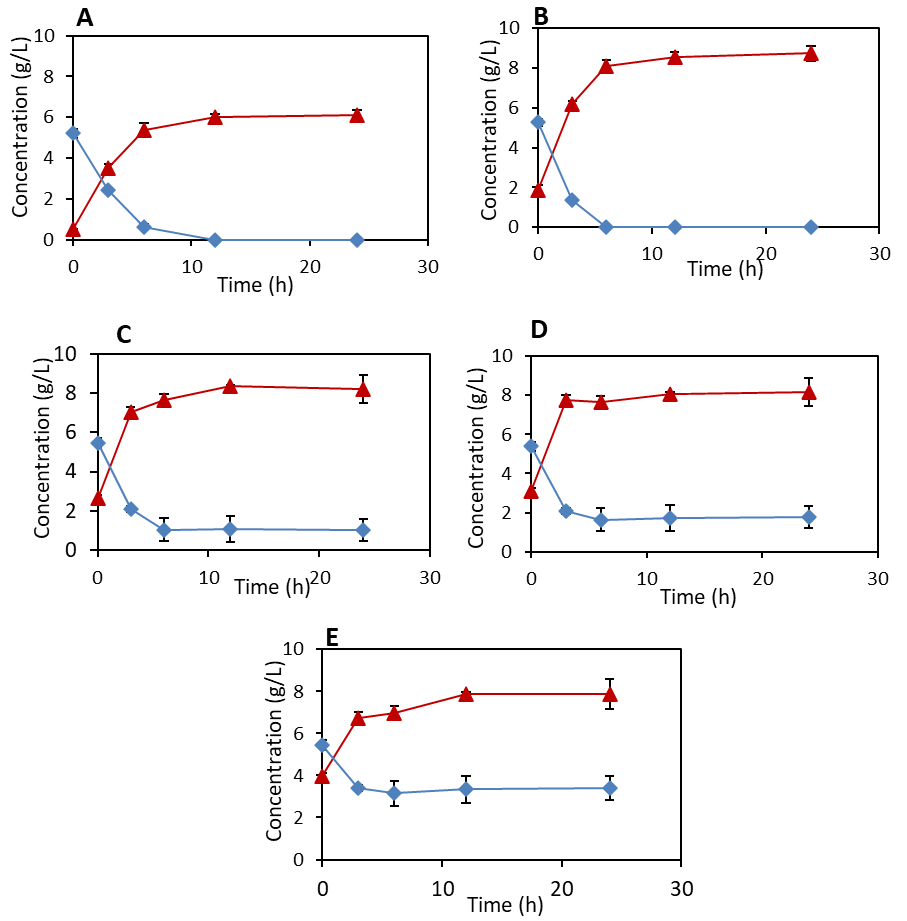


**Figure S6**. Oxidation of 5 mg/mL FFCA (♦) to FDCA (▲) in the presence of FDCA at concentrations of (A) 0 mg/mL, (B) 2 mg/mL, (C) 5 mg/mL, (D) 7.5 mg/mL, and (E) 10 mg/mL in 0.1M acetate buffer pH 5 at 30 °C using 52 mg wwt/mL resting cells of *G. oxydans* DSM 50049.

**Table S1.** Primer sequences for gene amplification using Phusion polymerase and its T_m_ / annealing temperature. T_m_ of paoA_F without restriction site is 65.2℃, and T_m_ of paoB_R without restriction site is 68.5 ℃. Restriction sites and overlapping sequences for Gibson assembly are indicated with underlined and bold italic text, respectively.

| Primer | Sequence (5’→3’) | T_m_ (℃) | Annealing temp. (℃) |
| --- | --- | --- | --- |
| ddmA1_F  ddmA1_R | CAAGCATATGGTGACCAGAAAGCCGGATAT  GACCAAGCTTGGCCTTTTCCTGCATGAAAC | 75.7  70.0 | 66 |
| adhB2_F  adhB2_R | GGCCCATATGCTGAACGCATTAACTCG  GGTGCTCGAGTTGTGCGTCGTCCA | 70.5  73.4 | 66 |
| 206_F  206_R | TAAGCACATATGGGCAGGACGCATGTC  TAATGACTCGAGCACCCGCCTGATTGACCC | 71.4  75.1 | 66 |
| 2677_F  2677_R | TAAGCACATATGGCTAAAATCGAACAGATTGCGAA  TAAGCAAAGCTTGCTCGTATGCTGCTGCAG | 70.9  73.4 | 66 |
| sldBA_2-F1  sldBA_2-R1  sldBA_2-F2  sldBA_2-R2 | CGCGGCAGCCATATGATCGCCGGG  ***CTGCGCATGACGGTTCCCCT***CAGG  ***GGGGAACCGTCATGCGCAG***ATCCCATCTTCTCGCC  TGGTGGTGCTCGAGTCAGGATGCCGCTCTGCGGGTCATAGGACACGGAA | 76.1  73.8  79.9  85.1 | 68 |
| 2764_F  2764_R  2763_F  2763_R | GCCGCGCGGCAGCCATATGGCGGAGGGACGTGCGCT  ***GACCCGATCATTCCCGACATA***ACGCAGG  ***TATGTCGGGAATGATCGGGTC***GCGGAC  TGGTGGTGGTGGTGCTCGAGTTATGACAGGATTGGGGCGAATGTAACA | 75.7  70.0  73.9  81.3 | 66 |
| calB_F  calB_R | TTACATATGTCCGATACTGTTTCC  GGCAAGCTTGTCAGAGGAGCCAGCGTA | 61.3  74.5 | 61 |
| 1307_F  1307_R | TTACATATGCTGTCGATGGCGCAG  GGCGCGGCCGCGTAGCTGTAGCCGTAGGA | 68.0  74.3 | 68 |
| tyrC_F  tyrC_R | TTACATATGATCTGCGTTCCCGTT  GGC**AAGCTT**GGCCTGACGGTTTTCAAT | 65.2  72.7 | 65 |
| paoA_F  paoA_R  pao575_F  pao575_R  paoB_F  paoB_R | GCCGCGCGGCAGCCATATGATGACCGATGTGTCTGTCGC  ***TGTCTCATTGCGGCT***TCTCCGGC  ***AGCCGCAATGCGACA***GTTCTCCTATTCCCGC  ***CCGATTTCATGATCGATGA***GATTGGTGCCCC  ***TCATCGATCATGAAATCGG***ACGTCGAACGAC  TGGTGGTGGTGGTGCTCGAGTCAGAAAATGGTCTTTTGCGTCTGGG | 82.5  72  77  72.9  72  81.4 | 70 |
|  |  |  | 70 |
|  |  |  | 69 |

**Table S2**. Conditions applied for amplification and cloning of the target genes from G. oxydans 50049 genome.

| No. | Gene | Annealing temp. (℃) | Conc. of  amplified gene (ng/μL) | Restriction enzyme | Conc. of gene in digestion (ng/μL) | Conc. of digested gene*^2^  (ng/μL) | Required molar mass for ligation*^3^ (ng) | Conc. after plasmid mini prep (ng/μL) |
| --- | --- | --- | --- | --- | --- | --- | --- | --- |
| 1 | ddmA1 | 66.2 | 33.5 | NdeI  HindIII | 26.8 | 33.5 | 31.49 | 29.5 |
| 2 | adhB2 | 66.2 | 38.0 | NdeI  XhoI | 30.4 | 38.0 | 52.07 | 58.21 |
| 3 | 206 | 66.2 | 36.9 | NdeI  XhoI | 29.52 | 36.9 | 44.32 | - |
| 4 | 2677 | 66.2 | 40.5 | NdeI  HindIII | 32.4 | 40.5 | 84.21 | 110.44 |
| 5-1 | sldA1 | 68 | 27.8 | NdeI  HindIII | 10.41^*1^ | - | - | 185.06 |
| 5-2 | sldA2 | 68 | 43.0 | NdeI  XhoI | 53.79^*1^ | - | - |  |
| 8 | 2764 | 66 | 52.72 | XhoI  NdeI | 20.27^*1^ | - | - | 199.42 |
| 9 | 2763 | 70 | 31.92 |  | 12.93^*1^ | - | - |  |
| 12 | caldh | 61 | 60.83 | NdeI  HindIII | 42.58 | 59.58 | 21.47 | 76.30 |
| 14 | tyrC | 65 | 27.38 | NdeI  HindIII | 20.53 | 10.09 | 16.72 | 117.16 |
| 15 | paoA | 70 | 48.0 | XhoI  NdeI | 17.22^*1^ | - | - | 289.14 |
| 16 | Pao575 | 70 | 23.9 |  | 3.761^*1^ | - | - |  |
| 17 | paoB | 69 | 57.87 |  | 30.32^*1^ | - | - |  |

^*1^ Required molar mass; unit is ng, used for Gibson assembly in a total volume of 20 μL.

^*2^ Concentration after gel purification of the digested gene.

^*3^ insert:vector ratio was 1:1 for all ligation and Gibson assembly.

**Table S3**. Tested conditions for the expression of the selected oxidoreductase genes.

| No. | Annotated Enzyme | Expression Host | Antibiotics | Medium | (Expression) / Conditions |
| --- | --- | --- | --- | --- | --- |
| 1 | FAD-dependent Oxidoreductase | BL21 | Kan | LB | (+) / 0.5 mM IPTG, 16 °C, overnight |
| 2 | Alcohol dehydrogenase (ADH) | BL21 | Kan | LB | (+) / 0.5 mM IPTG, 16 °C, overnight |
| 4 | Membrane-bound aldehyde dehydrogenase (MALDH) | BL21 | Kan | LB | (+) / 1 mM sodium molybdate  0.2 – 0.7 mM IPTG, 16 °C, 20 hours  4 mg/mL L-arabinose (GroEL and GroES) [1] |
| 8 | Xanthine dehydrogenase | BL21-CodonPlus | Kan, Cm, Strep | LB | (+) / 1mM sodium molybdate, 0.5 mM IPTG, 16 °C, 20 hours |
|  |  |  |  | AI | (-) / 37 °C, 5h, 16 °C, 20 hours |
| 12 | Coniferyl aldehyde dehydrogenase (CALDH) | Bl21-CodonPlus | Kan, Cm, Str | LB | (+) / 0.5 mM IPTG, 16 °C, 20 hours, 4 mg/mL L-arabinose (GroEL and GroES) |
|  |  |  |  | AI | (+) / 37 °C, 5 hours, 16 °C, 20 hours |
| 14 | Cyclohexadienyl dehydrogenase | BL21 | Kan | LB | (+) / 0.5 mM IPTG, 37 °C, 4 h, 4 mg/mL L-arabinose (GroEL and GroES) |
|  |  |  |  | AI | (+) / 37 °C, 5 hours, 16 °C, 20 hours |
| 15 | Aldehyde oxidoreductase iron-sulphur-binding | BL21 | Kan | LB | (+) / 0.5 mM IPTG, 16 °C, 20 hours |
|  |  |  |  | AI | (-) / 37 °C, 5 hours, 16 °C, 20 hours |
| Abbreviations: LB: Luria-broth; AI: auto-induction medium; Kan: Kanamycin; Cm: Chloramphenicol; Str: Streptomycin; GroEL: protein belonging to the chaperonin family of molecular chaperones, required for the proper folding of many proteins in bacteria; GroES: co-chaperonin protein that helps GroEL to function properly.  *The numbers in the first column are taken from Table S2.  BL21-CodonPlus is Cm and Str resistant. | | | | | |

**Figure S7.** Nucleotide sequence of the selected genes from G. oxydans DSM50049 and the corresponding amino acid sequences.

**Figure S8.** SDS-PAGE analysis of the five enzymes where **(1)** is FAD-dependent oxidoreductase (30.9 kDa), **(12)** is Coniferyl aldehyde dehydrogenase (31.3 kDa), **(2)** is Alcohol dehydrogenase (51.1 kDa), **(4)** is Membrane-bound aldehyde dehydrogenase (83.1 kDa), **(8)** is Xanthine dehydrogenase (25.3 kDa), **(14)** is Cyclohexadienyl dehydrogenase (24.1kDa), **(15)** is Aldehyde oxidoreductase iron-sulphur-binding (54.8 kDa) and the purification attempt. The legends are written as: Number, corresponding to the enzymes as indicated in Table S3, the strain and medium used for enzyme production. The molecular sizes of the enzymes are written at the bottom of the gel. Abbreviations, I: insoluble fraction, S: soluble fraction, F: purified enzyme fractions, AI: autoinduction medium. The produced targeted enzymes are indicated with white arrows.


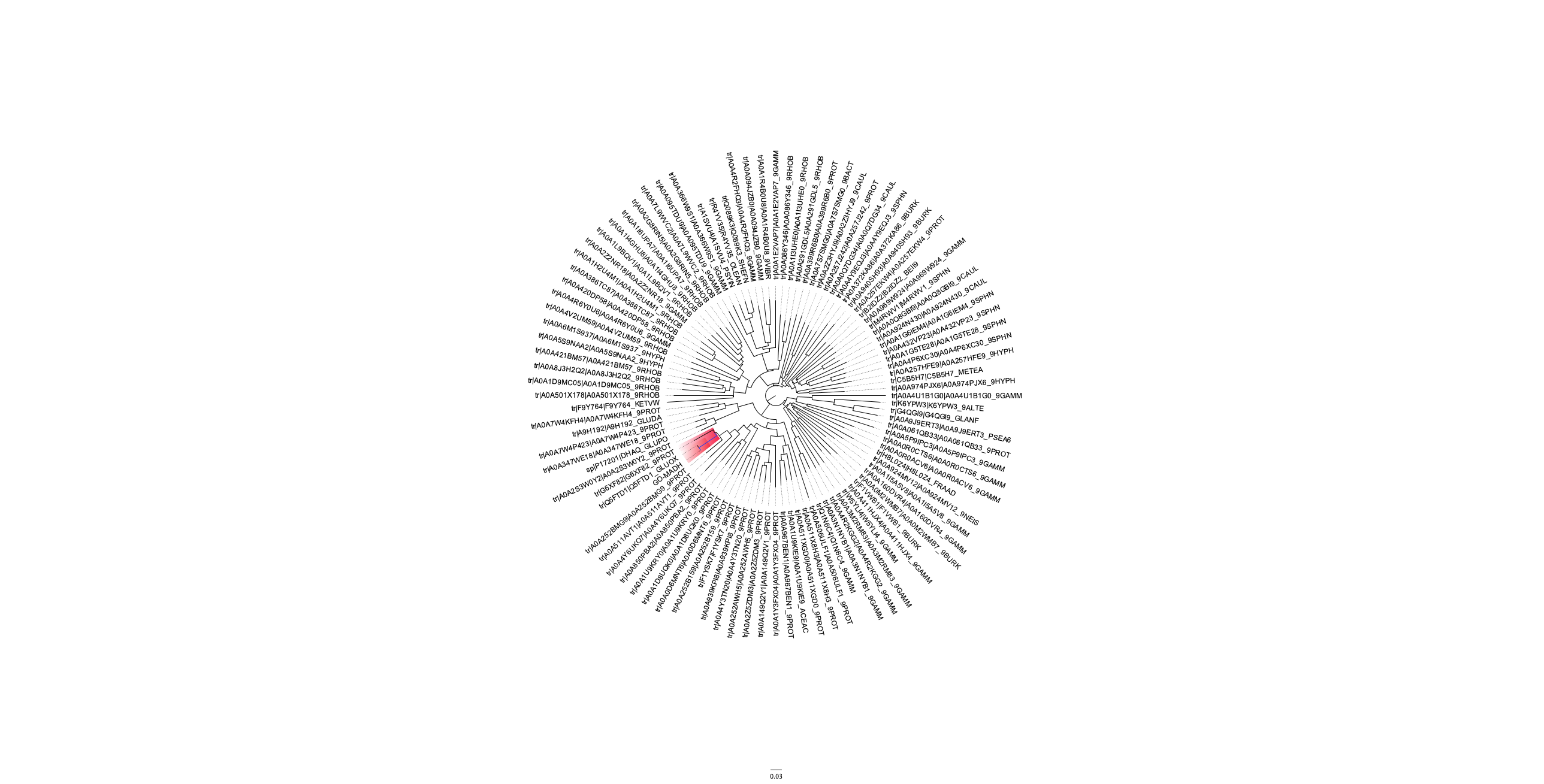


**A**


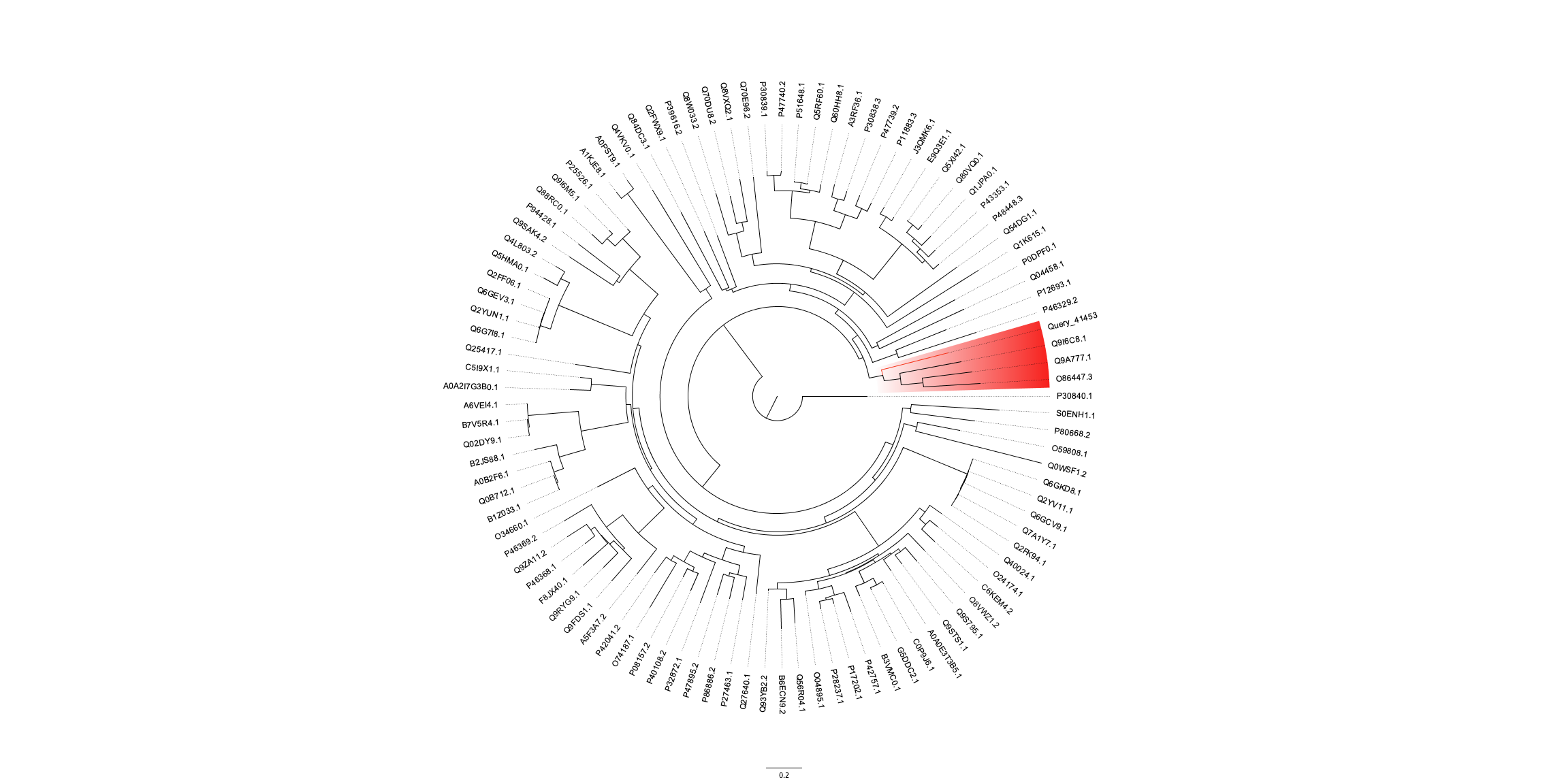


**B**

**Figure S9.** Phylogenetic tree generated using: (A) UniprotKB reference proteome and Swiss-Prot for membrane bound aldehyde dehydrogenase from *G. oxydans* DSM 50049 (GO-MADH, red line); the other related PQQ dependent dehydrogenases are highlighted in red, and (B) blast pairwise alignments employing Non-redundant UniProtKB/Swiss-Prot sequences database for the target CALDH from *G. oxydans* DSM 50049 (Query 41453, red line); the target protein is related to other CALDH enzymes as indicated from the red highlighted part of the tree.


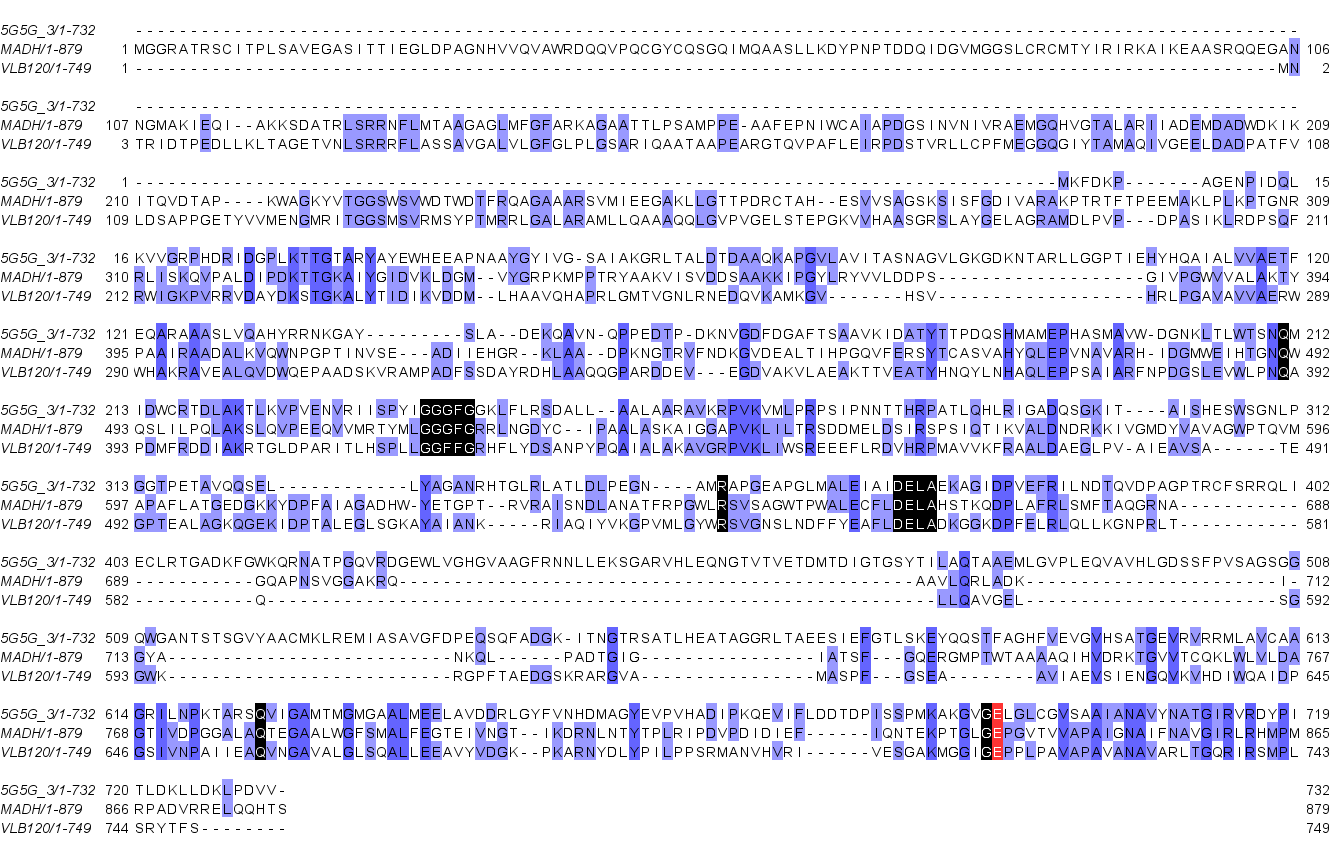


**Figure S10**. Protein sequence alignment of MALDH, aldehyde oxidase from Pseudomonas sp. VLB120 (A0AAN0R257), and aldehyde oxidase from E. coli (PDB:5G5G). Highlighted in black with white font are pterin cytosine dinucleotide (MCN) and dioxothiomolybdenum (VI) (MOS) binding sites, while active site residue is highlighted in red font. The conserved residues in the three enzymes are indicated in dark blue. Alignment was made using ClustalW.


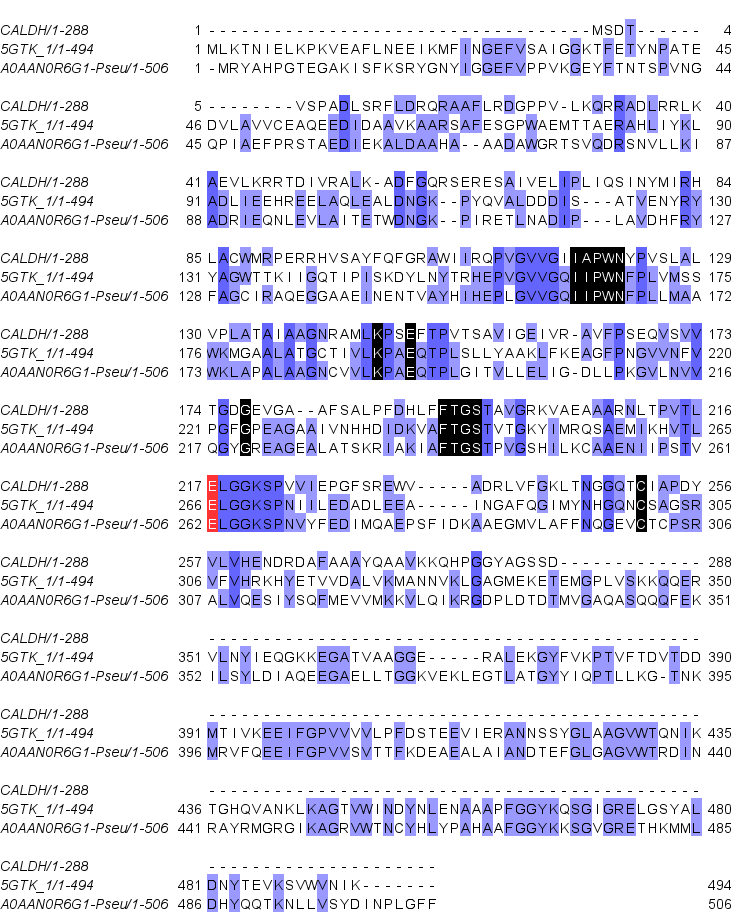


**Figure S11**. Protein sequence alignment of CALDH with NAD^+^ dependent aldehyde oxidase from Pseudomonas sp. VLB120 (A0AAN0R6G1) and NAD^+^ dependent aldehyde oxidase from Bacillus cereus (PDB: 5GTK). NAD^+^ binding site is highlighted in black while active site residue in red. The conserved residues in the three enzymes are indicated in dark blue. Alignment was made using ClustalW [2].

**References**

1. Nishihara K, Kanemori M, Yanagi H, Yura T: Overexpression of trigger factor prevents aggregation of recombinant proteins in *Escherichia coli*. Appl Environ Microbiol. 2000, **66:**884-889.

2. Madeira F, Madhusoodanan N, Lee J, Eusebi A, Niewielska A, Tivey ARN, Lopez R, Butcher S: The EMBL-EBI Job Dispatcher sequence analysis tools framework in 2024. Nucleic Acids Res. 2024, **52:**W521-W525.
